# Standardizing characterization of membrane active peptides with microfluidics

**DOI:** 10.1063/5.0048906

**Published:** 2021-07-07

**Authors:** Kareem Al Nahas, Ulrich F. Keyser

**Affiliations:** Cavendish Laboratory, University of Cambridge, J.J. Thomson Avenue, Cambridge CB30HE, United Kingdom

## Abstract

Antimicrobial peptides (AMPs) are emerging as important players in the fight against antibiotic resistance. In parallel, the field of microfluidics has matured and its benefits are being exploited in applications of biomimetics and standardized testing. Membrane models are essential tools extensively utilized in studying the activity and modes of action of AMPs. Here, we describe how the utilization of microfluidic platforms in characterizing membrane active peptides can develop a reliable colorful image that classical techniques have rendered black and white.

## I. INTRODUCTION

**FIG. 1.**
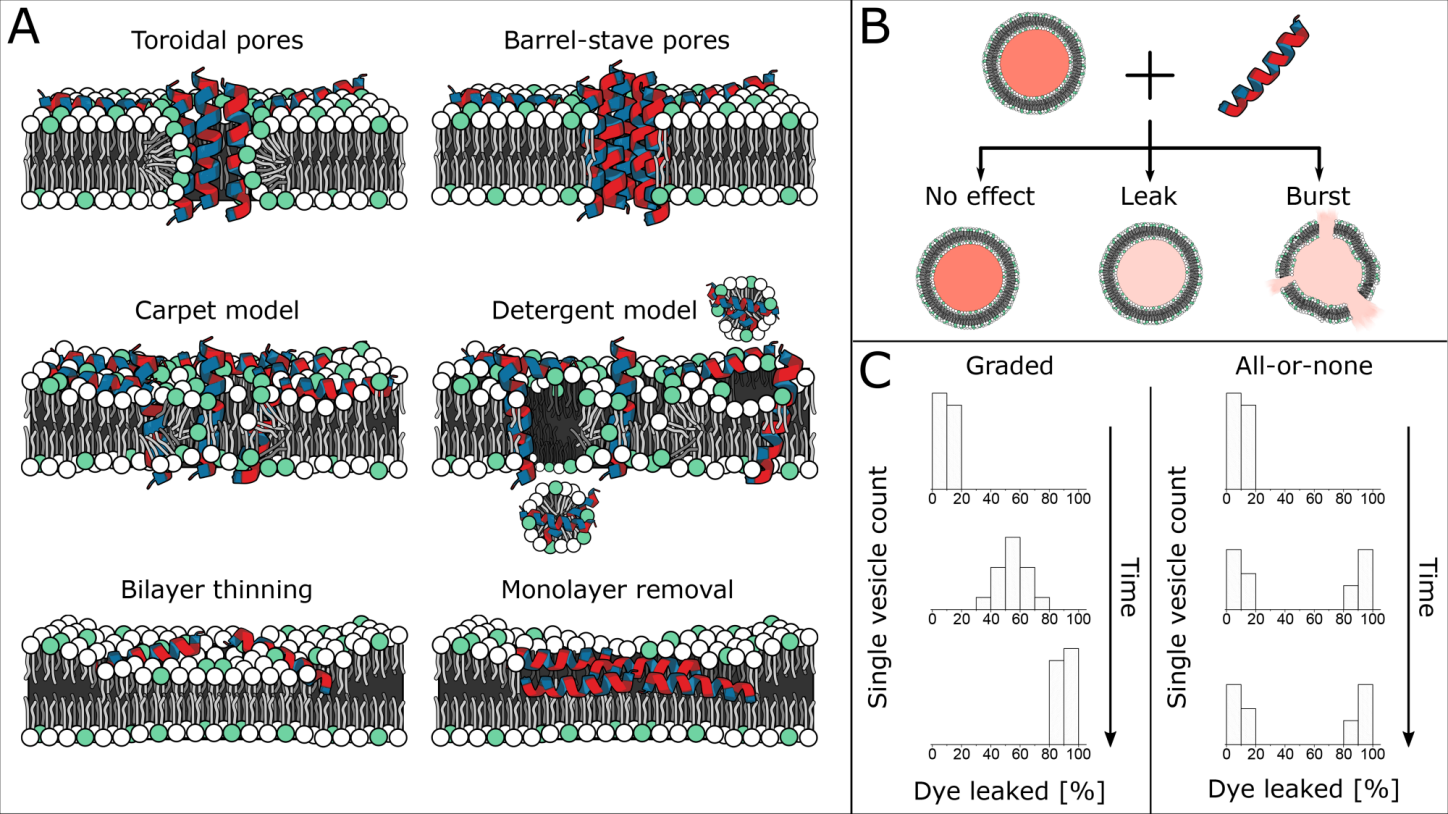
Schematics depicting typically cited AMP mechanisms of bilayer disruption, peptide-vesicle observed behaviors, and the modes of action at the population level. (a) Toroidal and Barrel-stave pores span the bilayer and lead to content leakage while maintaining membrane integrity. The main feature in the carpet model is the “carpeting” of the bilayer with peptides until a critical concentration is reached, which can lead to pore formation or detergent-like disruption. Membrane thinning or monolayer removal typically occurs when peptides initially bind to the bilayer. (b) Dye-loaded vesicles when treated with AMPs can exhibit complete membrane disruption, content leakage, or no observable effect. (c) At the population-level, peptide treated vesicles can behave throughout time in a graded manner where the entire vesicle population acts uniformly or in an all-or-none manner, where the population splits into two subpopulations of intact and non-intact vesicles.

Antimicrobial peptides (AMPs) have been heavily promoted as a promising class of antibiotics that can circumvent typical resistance mechanisms.^1^ This is due to the inherent ability of AMPs to disrupt microbial cell membranes. Over the years, the use of model lipid membranes has offered access to a wealth of valuable information on whether a peptide can disrupt lipid bilayers and potentially possess antimicrobial properties. According to the literature on model membranes, numerous theories by which peptides disrupt a lipid bilayer have been suggested.^2^ Nevertheless, the exact models describing the modes of action of peptides are the subject of much debate, and the nature of membrane pores formed by peptides is yet to be fully elucidated.^1,3^ Some of the main classes of membrane disruption theorized include pore formation (toroidal and barrel-stave), carpet model, membrane thinning, and possibly a combination of modes [Fig. 1(a)]. A peptide’s sequence has an important role in defining its membrane disruption mechanism, though it is widely assumed that any cationic peptide can demonstrate antimicrobial activity.^4^ Membrane disruption is strongly dependent on peptide concentration; passing a concentration threshold is typically necessary to exhibit membrane disruption^5^ or the switching of a peptide’s activity from one mechanism to another.^6^ An additional layer of complexity, when studying membranolytic peptides, is the observed all-or-none vs the graded leakage phenomenon [Fig. 1(c)].^7^ It is suggested that the graded mode subscribes to the equilibrium model, where the tested vesicle population falls under a unimodal distribution describing equal leakage of all individual vesicles. Conversely, the all-or-none mode is believed to be caused by stochastic interactions and exhibits bimodal distributions describing subpopulations of intact and leaked vesicles. In ensemble measurements, the all-or-none mode can be confused with the graded partial transient leakage mode, since both modes can describe the same leakage trace. The latter has been reported as transient observations entailing a burst of vesicle leakages occurring immediately after contact with a peptide, followed by cessation of observed leakage.^3,8^ In essence, characterizing the membranolytic activity of peptides is not trivial and has challenged researchers, who have had to rely on multiple assays to build an understanding of the issues at play.

## II. BOTTOM-UP: ENSEMBLE TECHNIQUES

The characterization of peptide-membrane interactions using ensemble techniques, such as bulk leakage assays, has been the bedrock for observing and quantifying AMP efficacy. The minimalism in lipid models can highlight the significant components and targets involved in AMP activity. Membrane models allow us to incrementally build up the complexity of the model, tailor lipid compositions, explore different physiological conditions, and reconstitute purified membrane proteins while introducing minimal biological noise. Ensemble membrane leakage assays are standardized assays, which typically utilize large unilamellar vesicles (LUVs) ranging between 100 and 1000 nm in diameter. The leakage assays can be easily parallelized in microtiter plates and adopted in almost any wet laboratory. Nevertheless, the simplicity of the experimental approach comes at the cost of observing the membrane behavior at the bulk level instead of the single vesicle level and as a consequence could lead us to make incorrect assumptions about AMP activity. When treating a lipid vesicle with AMPs, we can expect one of three scenarios—the vesicle may remain unaffected, or it could leak while the membrane remains intact, or it could burst and release its contents, with complete lysis of the membrane [Fig. 1(b)]. Bulk vesicle leakage assays can provide neither direct information about the disruption mechanism involved nor the mode in which a vesicle population could have behaved, as it does not reveal individual events. Furthermore, the vesicle ensemble measurements are prone to vesicle aggregation and fusion, as anionic LUVs are mixed with cationic peptides freely.^7^

## III. FREEHAND GUV TECHNIQUES

The move to quantitative imaging techniques has become essential in revealing the mechanistic information for peptide activity hidden at the single vesicle or cell level. When it comes to lipid models in the microscopic range, giant unilamellar vesicles (GUVs) are key, with diameters ranging between 1 and 100 μm.^9^ Several studies have been conducted using GUVs to elucidate and confirm the modes of action and mechanisms of antimicrobial peptides.^9–11,43^ Distinguishing leakage mechanisms optically is achieved by monitoring the states of a vesicle’s contents and its lipid membrane (Fig. 1). For example, pore formation corresponds to observing content leakage while the lipid membrane is intact, as opposed to content leakage due to membrane lysis as in the case of the detergent model. Differentiating barrel-stave pores from toroidal pores is typically investigated by studying the rate of lipid flip-flop between the two leaflets of the lipid bilayer. Here, toroidal pores correspond to a high rate of lipid scramble, while the scrambling abilities of barrel-stave pores are minimal.^12,13^ The carpet model is distinct because it requires a critical concentration of membrane bound peptides to be reached before a leakage event takes place. With regards to membrane thinning or monolayer removal, these mechanisms do not display clear distinguishing leakage features at the single vesicle level as vesicles can remain intact; these require other complementary techniques like in-liquid AFM images of supported lipid bilayers for visualization.^14^ Single vesicle resolution studies mainly focused on the monitoring of leakage kinetics or the observation of stochastic all-or-none and graded partial leakage modes of action as they can easily determine the two modes, observations bulk measurements cannot directly make.^15,16^ These studies either relied on measuring the leakage at high temporal resolution for a handful of vesicles or looked at populations at a single time point after peptide incubation.^10,17^ Freehand analysis of individual GUVs from microscopy images is the basis for studies where vesicles are unconfined in a bulk environment and require extensive manual handling, rendering the standardization of these assays challenging. Peptide dosing methodology also varies either by adding vesicles into the peptide solution and missing out on initial disruption kinetics or diluting the candidate peptide into the settled vesicles, relying on diffusion, which results in varying the effective local peptide concentration over time. Kinetic leakage studies require constant attention, especially when the GUVs are not immobilized and are prone to movement during measurements over long time periods. Generally, freehand GUV studies lack control and suffer from having to compromise on spatiotemporal resolution over throughput or vice versa.

## IV. MICROFLUIDIC GUV TECHNIQUES

The mentioned drawbacks are overcome by transitioning from bulk into microfluidic environments. The field has built and characterized an arsenal of microfluidic tools for biological research. By integrating various established tools, researchers can develop bespoke “total analysis” systems that accommodate their own research questions. In the case of membrane permeabilization studies, microfluidics can offer high-throughput solutions for GUV formation, immobilization, multiplexing, and highly controlled liquid exchange. In addition, the minimal use of valuable compounds in microfluidic devices is an important factor to consider in comparison to the large quantities needed in bulk studies. Therefore, we are advocating for microfluidics-enabled reliable and multifaceted solutions for standardized peptide characterization assays, which can also be automated as complementary approaches to established techniques.

### A. Giant vesicle formation

**FIG. 2.**
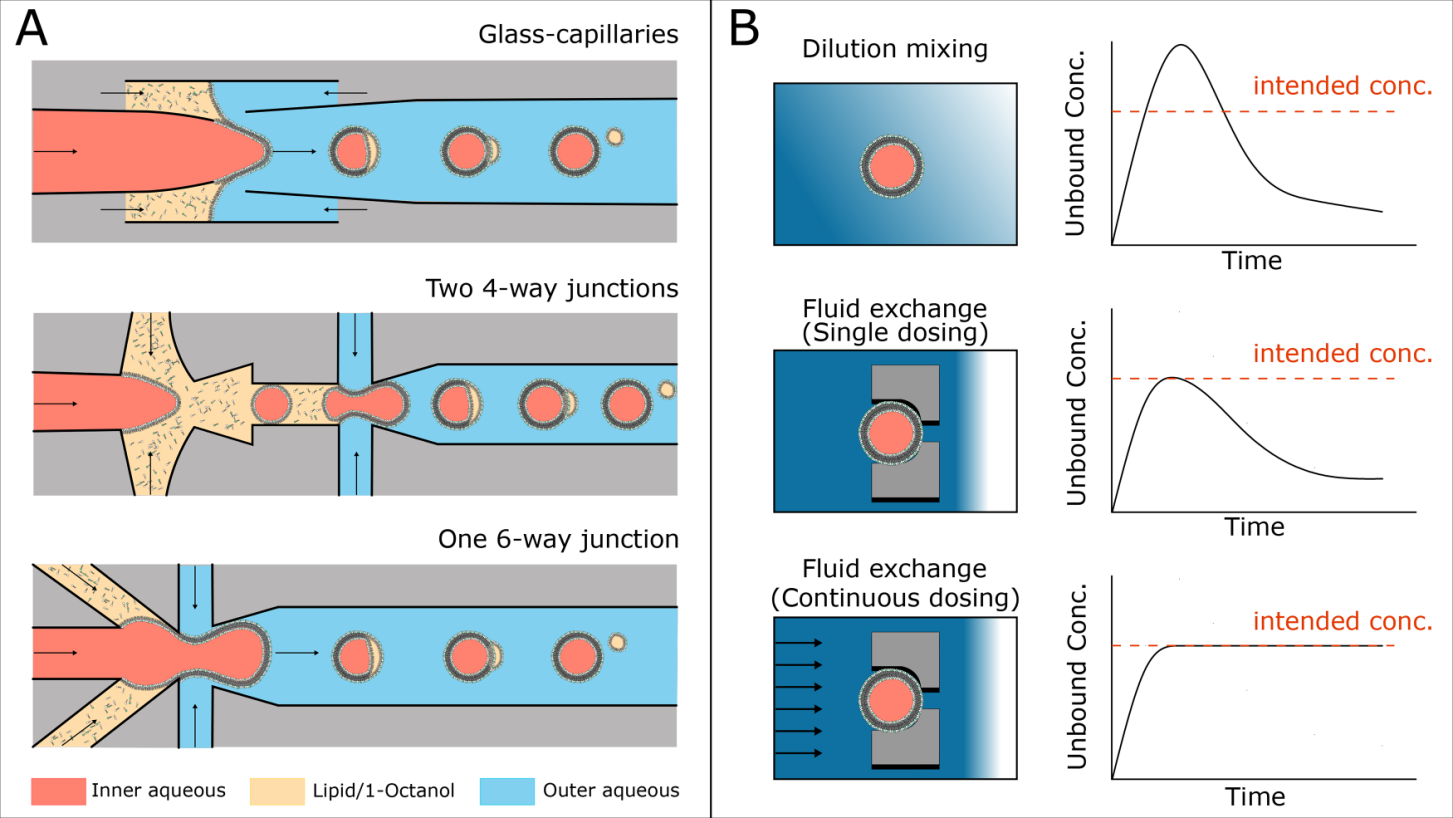
Schematics depicting (a) three mainstream microfluidic systems for generating water-in-oil-in-water (W/O/W) double emulsions as a template toward GUVs. GUV formation process in glass-capillaries, and two Polydimethylsiloxane (PDMS) based techniques involving either two consecutive four-way junctions or a single six-way junction.^18–20^ (b) Three approaches for introducing peptides into populations of immobilized vesicles and correlating hypothetical concentration profiles of the local unbound peptides in the vicinity of the vesicles. Dilution mixing entails pipetting a few microliters of a highly concentrated peptide into an open chamber/well and relies on diffusion for peptide exposure. Fluid exchange entails total exchange of a trapped vesicle’s surrounding fluids in a microfluidic chamber with fluids containing the intended peptide concentration. The flow can be stopped shortly after perfusion to achieve the effect of a single dose, or flow can continue to deliver a fresh solution and maintain control over the surrounding peptide concentration regardless of binding.

The literature provides a large number of giant vesicle generation protocols.^21,22^ Nonetheless, the optimization and development of novel techniques is still in flux. This indicates that an ultimate process that can bypass all the drawbacks with the current techniques is yet to be obtained. The desired standards in GUV preparation include unilamellarity, uniformity and homogeneity, high yield and throughput, flexibility with salt concentrations, high encapsulation efficiency of large and charged molecules, solvent and detergent free membranes, easy size control, as well as rapid and reliable usage. The most well-established and commonly used methods are electroformation, spontaneous swelling, and inverted emulsion. In recent years, microfluidics has gained attention in the formation of water-in-oil-in-water (W/O/W) double emulsions containing lipids in the oil phase (Fig. 2). The microfluidic techniques are bypassing many of the disadvantages found in the more established techniques. The choice of protocol is usually made by utilizing the existing expertize in the research group and the type of study to be conducted. For studying AMPs at the vesicle population level, we recommend the use of microfluidic based techniques as they enable high-throughput production of uniform GUVs (tens to thousands of Hz) on demand and can easily encapsulate homogenously charged membrane impermeable dyes as membrane integrity markers at physiological salt conditions.^18–20^ There are various optimizations and characterizations possible in microfluidics-based GUVs manufacturing to overcome drawbacks, such as the presence of stabilizers and additives, which may give microfluidic techniques an edge in reaching the gold-standard of giant vesicle preparation.^23–25^

### B. Vesicle trapping and peptide administration

Vesicle trapping serves as a twofold solution when it comes to conducting AMP studies. The first function is to immobilize GUVs for imaging over prolonged periods of time and to conduct high spatiotemporal resolution studies. The second function is to enable the total exchange of the vesicle’s surrounding fluids, giving control in defining the initial time of peptide-vesicle exposure and the concentration of the peptide at the point of contact. In bulk studies, rapid and homogeneous fluid exchange around vesicles is challenging and peptide administration is mainly subject to dilution mixing. This means that bulk conditions can vary continuously depending on the distance between exposed vesicles and the point of injection. Initial peptide concentrations can be at higher levels than intended, causing a system shock before reaching equilibrium or lower levels of available peptides than intended due to binding [Fig. 2(b)].

In a recent study, pharmokinetic dosing of a peptide using an *in vivo* model of infection has been shown to be an important factor in a peptide’s efficacy and toxicity.^30^ Here, a comparison has been made between single injections vs continuous perfusion, indicating improved efficacy and mouse survival with continuous subcutaneous administration over single dosing schemes.^30^ The reported decay in peptide concentrations over time after single dosing gives similar impressions to the transient model reported in bulk leakage experiments. We argue that peptide administration in classical studies is overlooked and can benefit from comparative controlled studies between continuous dosing and single dosing on vesicle and bacterial populations.

When it comes to vesicle immobilization, the case is similar to vesicle preparation, as there is an array of techniques and trap designs that can be utilized.^31^ When choosing a method to conduct GUV permeabilization studies, trapping standards to consider include throughput, spatial control, additives and modifications, and swift fluid exchange under minimal shear stress. Accordingly, the simplest immobilization method is surface tethering, most commonly using biotin-avidin binding, coupled with a microfluidic channel for swift fluid exchange.^32^ This comes with the caveat of having to introduce modifications to the lipids, and the risk of adhesion affecting membrane properties. A more spatially controlled, functionalization-free approach is the use of microstructures that can trap GUVs under flow. Hydrodynamic traps often consist of two micro-posts with a small gap in between that can hold a single GUV [Fig. 2(b)]. A large array of these traps can be placed in a microfluidic chamber to observe the population’s behavior under the same conditions.^33^ The traps can be coupled with individual donut shaped valved compartments that can create local static environments for the trapped vesicles.^34^ This prevents the replenishment of membrane bound peptides after initial exposure and can mimic single dosing kinetics [Fig. 2(b)]. Introducing more global valve solutions at the chamber level can also serve to decrease the complexity in manufacturing while maintaining high trapping throughput. The above-mentioned approaches have shown their value for conducting controlled fluid exchanges, and such devices can be used to study the effect of peptide dosing under static or continuous flow conditions.

### C. Microfluidic multiplexing and integration

Conducting studies in parallel enhances the efficiency of peptide testing assays as it provides more data per experimental run under standardized conditions. In bulk GUV assays, simultaneous handling and assaying of multiple peptides at different experimental conditions can be laborious and time consuming. Microfluidic platforms have the inherent potential for multiplexing and running numerous peptides on populations of vesicles prepared from the same batch simultaneously. This can occur in multiple separate observation chambers,^29^ or using separately controlled arrays of valves each dedicated to a subset of trapped vesicles in a single chamber.^27^ In principle, the mentioned platforms have the precursor that enables the high-throughput screening of peptides and a manifold of important experimental parameters. Nevertheless, in the case of spatially defined GUV experiments, the throughput of microscopy based microfluidic screening assays are currently limited by the imaging solutions employed; therefore, compact microscope-arrays and feasible high-throughput imaging technologies play a major role in reaching high-throughput screening.^35^

**FIG. 3.**
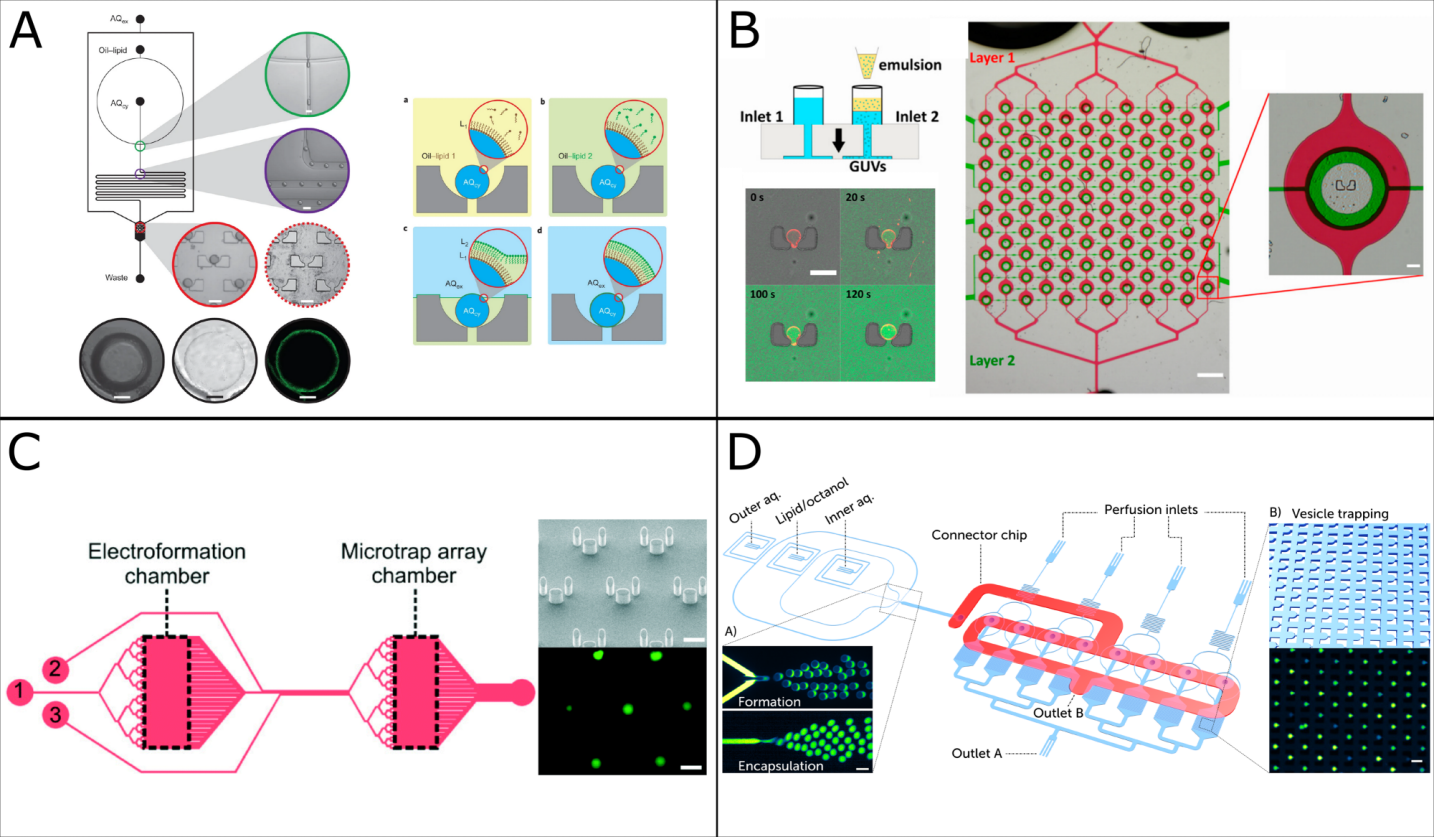
Collage summarizing four distinct microfluidic platforms that have integrated both vesicle formation and trapping capabilities in a single chip. Overview of (a) a layer-by-layer vesicle formation method that utilizes an array of two-post traps for bilayer assembly in a chamber.^26^ Adapted from Matosevic *et al.*, Nat. Chem. **6**(10), 1469–1482 (2013). Copyright 2013 Springer Nature. (b) Inverted emulsions method integrated with an array of tailored, valved microfluidic traps that possess serial multiplexing capabilities.^27^ Adapted from Yandrapalli *et al.*, Micromachines **11**(3), 285 (2020). Copyright 2020 MDPI, licensed under a Creative Commons Attribution 4.0 International License. (c) Electroformation chamber on chip connected to a chamber with trap arrays.^28^ Adapted from Paterson *et al.*, Lab Chip **14**, 1806–1810 (2014). Copyright 2014 the Royal Society of Chemistry, licensed under a Creative Commons Attribution 3.0 Unported License. (d) Octanol-assisted liposome assembly (OLA) technique linked to eight chambers each containing an array of traps that possess parallel multiplexing capabilities.^29^ Adapted from Al Nahas *et al.*, Lab Chip **19**, 837–844 (2019). Copyright 2019 the Royal Society of Chemistry, licensed under a Creative Commons Attribution 3.0 Unported License.

Integrating on-chip GUV formation and multiplexed GUV trapping arrays in a single platform is key for automation and standardization. The proposed integration is the first step in eliminating manual GUV handling between steps, a critical prerequisite for automation. Another advantage is simultaneous trapping during vesicle formation; hence the formation can be stopped once the traps are filled and experiments can commence subsequently. To our knowledge, there have been four published platforms integrating on-chip formation and vesicle traps (Fig. 3), each utilizing a different GUV formation methodology, two of which possess multiplexing capabilities.^26–29^ Despite their existence, it seems like the added complexity in microfabrication of these elaborate devices and the required expertize for operating them are a barrier in adopting these technologies outside their original laboratories. Integration enables automating device operation, which in turn alleviates the challenges of usage. Multiplexing significantly expands on these devices’ throughput and the combination of both could incentivize further utilization in the wider community.

### D. Importance of studying many GUVs

While studying a single peptide at a single concentration treating a population of vesicles under controlled perfusion conditions, one might naively assume that every vesicle will behave in the same way and get disrupted at the same time following the equilibrium model. However, this is not always the case, as can be observed in the framework of all-or-none peptides. The platform from the Cooper laboratory [Fig. 3(c)] was used to characterize Magainin II and Melittin on populations of tens of trapped electroformed GUVs per experiment on chip.^36^ By monitoring leakage kinetics, they inferred different mechanisms of leakage at the population level, ranging from the carpet model, pore formation, bursting and vesicles surviving the peptide treatment.^36^ Due to the utilization of electroformation as the method of GUV generation in their platform, characterizing peptides under physiological salt concentrations was not possible. In our platform [Fig. 3(d)], we focus on populations of hundreds of anionic giant vesicles at physiological salt concentrations and the time distribution of vesicle leakage events in relation to different peptide concentrations. When testing Cecropin B and a series of *de novo* synthesized peptides, we see subpopulations of vesicles deviating from the mean time point of the population’s disruption events.^29,37^ Explanations of stochastic vesicle behavior and heterogeneities in event types and times are usually the subject of speculation, as the processes are not fully understood. Hence, to avoid drawing incomplete conclusions from small datasets, increasing the size of the vesicle populations while maintaining single-GUV resolution is paramount for analyzing event distributions, which can in turn lead us to a deeper understanding of the disruption mechanisms. Hence, we emphasize the importance of measuring the behavior of at least 200–600 GUVs, not only to distinguish between graded vs all-or-none modes of action but also to ensure the high statistical significance of the measurement and avoid mischaracterizing a peptide with a single mechanism.

## V. TOP-DOWN: SINGLE CELLS IN MICROFLUIDICS

In conjunction to model membrane bottom-up assays, top-down techniques are critical for studying AMPs and making correlations between the two approaches. Standard bacterial minimum inhibitory concentration (MIC) assays belong in the category of ensemble techniques and hence share many of the advantages and disadvantages discussed above. The accuracy of MIC assays is subject to the inoculum effect, as the efficacy of AMPs may vary due to the cell densities used.^38^ Varying cell densities can affect the concentration of unbound peptides, hindering cell-bound peptides from reaching the necessary threshold for killing a cell.^39^ This causes fluctuations in reported active peptide concentrations and “one-off MIC” values.^40^ Reasons for the described fluctuations can be the depletion of unbound peptides over time, various aggregation effects, accumulation of peptides in dead cell membranes and collation of peptides to bacterial DNA.^38–40^ Such factors cannot be easily observed or avoided in simple bulk assays.

Single-cell microfluidics can be used to replace bulk MIC assays for testing peptide efficacy. For example, the “Mother Machine” microfluidic device has recently been repurposed into a “Killing Machine” for testing the antibacterial activity of peptides and other antibiotics.^41^ The device has thousands of micrometer-sized dead-end channels to trap individual bacteria, all connected to a main channel that enables continuous fluid exchange. The platform has all the advantages outlined earlier in detecting the heterogeneity in bacterial behavior, in addition to studying persisters.^41,42^ It enables the continuous dosing of peptides at controlled concentrations, circumventing concentration dependent inoculum effects and offering individual cell tracking of growth changes while including high-throughput and multiplexing potential. The “Killing Machine” in combination with the GUV platform opens a pathway for comparable MIC studies, as the active concentration of antimicrobials can be directly compared. The additional data may help us clarify which mode of membrane disruption is predominant in cells, GUVs, or both. The first results from combinations of such approaches are just starting to be published.^37^

## VI. CONCLUSION AND OUTLOOK

Over the past years, the microfluidics community has produced a great number of techniques that enable the manipulation of GUVs and cells in ways not previously possible. The standardized characterization of peptides using controlled bottom-up and top-down microfluidic methodologies can provide a clearer picture in regard to peptide modes of action at the population level and the role of AMP administration strategies in terms of efficacy and toxicity. Matching the throughput of microfluidics with continuous fast confocal microscopy and integrated on-chip impedance measurements can expand our sensing capabilities and unravel hidden insights into membrane active peptides. We see a future where the standardization and throughput of the described microfluidics is utilized to the fullest in creating a large, reliable characterization database containing the most well-known AMPs. After verification, this can be expanded to any peptide, and the enhanced quality and reliability of such a database will be of critical importance in designing, categorizing, and ranking future AMPs.

## Data Availability

Data sharing is not applicable to this article as no new data were created or analyzed in this study.
